# Very small embryonic-like stem-cell optimization of isolation protocols: an update of molecular signatures and a review of current *in vivo* applications

**DOI:** 10.1038/emm.2013.117

**Published:** 2013-11-15

**Authors:** Dong-Myung Shin, Malwina Suszynska, Kasia Mierzejewska, Janina Ratajczak, Mariusz Z Ratajczak

**Affiliations:** 1 Department of Biomedical Sciences, University of Ulsan College of Medicine, Seoul, Korea; 2 Stem Cell Institute at James Graham Brown Cancer Center, University of Louisville, Louisville, KY, USA

**Keywords:** VSEL, Igf2-H19 locus, Oct4, Sall4

## Abstract

As the theory of stem cell plasticity was first proposed, we have explored an alternative hypothesis for this phenomenon: namely that adult bone marrow (BM) and umbilical cord blood (UCB) contain more developmentally primitive cells than hematopoietic stem cells (HSCs). In support of this notion, using multiparameter sorting we were able to isolate small Sca1^+^Lin^−^CD45^−^ cells and CD133^+^Lin^−^CD45^−^ cells from murine BM and human UCB, respectively, which were further enriched for the detection of various early developmental markers such as the SSEA antigen on the surface and the Oct4 and Nanog transcription factors in the nucleus. Similar populations of cells have been found in various organs by our team and others, including the heart, brain and gonads. Owing to their primitive cellular features, such as the high nuclear/cytoplasm ratio and the presence of euchromatin, they are called very small embryonic-like stem cells (VSELs). In the appropriate *in vivo* models, VSELs differentiate into long-term repopulating HSCs, mesenchymal stem cells (MSCs), lung epithelial cells, cardiomyocytes and gametes. In this review, we discuss the most recent data from our laboratory and other groups regarding the optimal isolation procedures and describe the updated molecular characteristics of VSELs.

## Introduction

The field of regenerative medicine is currently searching for a reliable source of pluripotent stem cells (PSCs) that could give rise to cells from all three germ layers.^1^ For almost 20 years, researchers have been attempting to harness embryonic stem cells (ESCs) that can be isolated from the embryos generated by *in vitro* fertilization^2, 3^ or therapeutic cloning.^4^ However, this strategy is burdened by ethical considerations. A promising source of PSCs can be generated by the genetic modification of adult tissues—induced PSCs^5, 6^—but this strategy is still under development and risks the formation of teratomas in the injected cells, in addition to rejection by the host immune system.^7^

Various potential types of adult stem and progenitor cells can now be isolated from bone marrow (BM), mobilized peripheral blood and umbilical cord blood (UCB) or derived from expanded *in vitro* cultures of adherent cells (such as mesenchymal stem cells (MSCs) and multipotent adult progenitor cells (MAPCs)) and are being investigated in clinical trials to determine their ability to regenerate damaged organs (for example, heart, kidney and neural tissues).^8^ Rare cases of chimerism after the infusion of unmanipulated donor BM, UCB or mobilized peripheral blood cells have been reported by some investigators; however, these results can be explained by cell fusion^9, 10^ or presence of rare populations of stem cells that are endowed with multi-tissue differentiation abilities.^8^

Thus, two of the most intriguing questions in stem cell biology are (1) if adult tissues contain PSCs or multipotent stem cells and (2) if these cells can differentiate into cells from more than one germ layer. Several groups of investigators have employed various isolation protocols, surface marker detection systems and experimental *in vitro* and *in vivo* models and have reported the presence of cells that possess pluripotent/multipotent characteristics in various adult organs. Such cells have been assigned various operational abbreviations and names in the literature, such as MAPCs,^11^ multipotent adult stem cells (MASCs),^12, 13^ unrestricted somatic stem cells,^14^ marrow-isolated adult multilineage-inducible cells^15^ and multilineage-differentiating stress-enduring stem (Muse) cells.^16^ However, this raises the basic question: are these truly distinct cells or instead just overlapping populations of the same primitive stem cell? In fact, taking into consideration the common features described in the literature, it is very likely that various investigators have described overlapping populations of developmentally early stem cells that are closely related. Unfortunately, these cells were never characterized side-by-side in order to address this important issue. Moreover, the rare and quiescent population of so-called very small embryonic-like stem cells (VSELs), which was initially isolated from murine tissues and human UCB by our group^17, 18^ (and subsequently confirmed by other laboratories^19, 20, 21, 22, 23^), expresses several PSC markers and, in addition, shares some characteristics with the abovementioned cell populations.

VSELs circulate in PB under steady-state conditions; however, the number of cells is very low. In our recent study, we provide evidence that VSELs can mobilize into PB in mice and adult patients who have been injected with granulocyte colony-stimulating factor.^24^ This observation laid the foundation for the concept that granulocyte colony-stimulating factor mobilization can be employed to harvest VSELs from patients for therapeutic purposes. Furthermore, our studies on VSEL mobilization into PB reveal that VSELs are mobilized not only in patients suffering from myocardial infarct^25^ and stroke^26^ but also in patients suffering from skin burns,^27^ active inflammatory bowel disease^28^ and cancer.^29^ In a recently published paper, Taichman *et al.*^19^ demonstrated that VSELs circulating in PB in tumor-bearing mice may give rise to tumor-associated fibroblasts involved in tumor expansion.^29^ More importantly, based on our preliminary data, we also believe that the number of VSELs circulating in PB could be prognostic of myocardial infarct or stroke.^25, 26^ This notion, however, requires further study and long-term clinical confirmation.

In this article, we discuss the most recent data from our laboratory and others on the optimization of VSEL isolation from adult tissues, provide an update on the molecular characteristics of VSELs, and describe the potential roles of these rare cells in regenerative medicine.

## The presence of potential pluripotent stem cells in adult tissues

For many years, it has been accepted that adult tissues contain only tissue-committed stem cells (TCSCs), such as epidermal, hematopoietic or skeletal muscle stem cells, which demonstrate a limited, single, lineage-restricted potential for differentiation. However, recent evidence demonstrates that BM-isolated cells may possess broader differentiation potentials than the hematopoietic stem cells (HSCs) or MSCs present in this tissue.^8^ If we exclude the possibility of stem cell plasticity and HSC transdifferentiation, one has to consider the possibility that BM contains other, more primitive populations of PSCs and multipotent stem cells that are deposited in BM during embryogenesis.

Accordingly, we consider two scenarios that could occur during early embryogenesis and the development of lineage-restricted TCSCs.^1^ In the first scenario, PSCs develop in the inner cell mass of the blastocyst, or later on in the epiblast, and after giving rise to more differentiated lineage-restricted TCSCs gradually disappear in the developing tissues. In contrast, in the second scenario—which we believe is more likely—some PSCs giving rise to TCSCs survive in developing adult tissues as a backup population of PSCs that renews the pool of TCSCs over time. In this scenario, PSCs are not only precursors of TCSCs during organ/tissue rejuvenation but also serve as a source of PSCs during emergency situations when these organs are damaged (for example, myocardial infarction and stroke). This scenario, however, requires the PSC population in adult tissues to be kept under control and in a quiescent state essential for preventing uncontrolled proliferation that could lead to the formation of teratomas. We believe that VSELs are a population of such cells, and the mechanism that keeps them quiescent in adult tissues is similar to the one described in migrating primordial germ cells (PGCs). This mechanism requires the epigenetic modification of the regulatory regions of some developmentally crucial genes that are, themselves, regulated by parental imprinting.^30^

Our recent research revealed that murine VSELs modulate the expression of parentally imprinted genes (for example, *Igf2-H19*, *RasGRF1* and *Igf2R* (insulin-like growth factor receptor 2)) via epigenetic changes, which may have an important role in insulin/insulin-like growth factor signaling (IIS).^31^ It is well known that imprinted genes have a crucial role in embryogenesis, fetal growth, the totipotential state of the zygote, and the pluripotency of developmentally early stem cells.^32^ Thus, modification of imprinting within the regulatory regions (that is, differentially methylated regions; DMRs) of these genes, which occurs in VSELs, is crucial for maintaining quiescence in the pools of these cells residing in adult tissues.^31, 33^ Accordingly, we observed that murine BM-sorted VSELs erase paternally methylated imprints within the DMRs of *Igf2-H19* and *RasGrf1*, whereas they hypermethylate the maternally methylated DMRs of *Igf2R* and *KCNQ1-p57*^*KIP2*^. As paternally expressed imprinted genes (*Igf2* and *RasGrf1*) enhance embryonic growth and maternally expressed genes (*H19, Igf2R* and *p57*^*KIP2*^) inhibit cell proliferation,^34^ the unique genomic imprinting pattern observed in VSELs represses the growth of these cells.^31^

**Figure 1:**
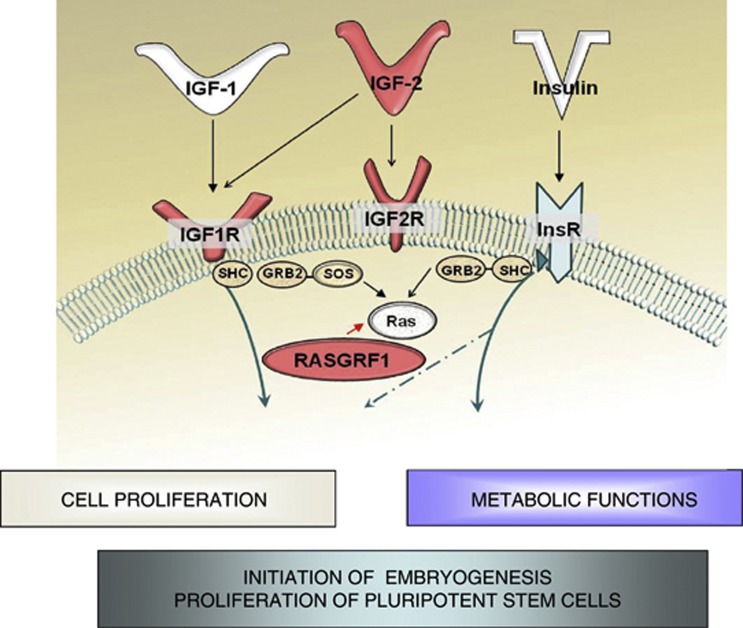
IIS signaling and imprinted genes. In mammals, there are three insulin factors (insulin, Igf1 and Igf2) that bind to two tyrosine kinase receptors (insulin receptor (InsR) and Igf1 receptor (Igf1R)). Igf2R is a non-signaling mannose-type sink receptor for Igf2. Activation of InsR and Igf1R leads to metabolic or proliferative responses depending on the cell type. RasGrf1 is a small GEF that is involved in signaling from InsR and Igf1R. VSELs demonstrate a decrease in Igf2 and RasGrf1 expression and the overexpression of Igf2R (shown in red) due to changes in the epigenetic state of the imprinted genes. These epigenetic changes in genes regulate IIS and maintain quiescence in VSELs in adult tissues that are somehow resistant to *in vitro* expansion. We hypothesize that chronic exposure to IIS accelerates the premature depletion of VSELs from adult tissues.

How do epigenetic changes in gene expression translate into changes in the state of VSELs? In murine VSELs (Figure 1), the abovementioned epigenetic changes lead to perturbation of IIS by downregulating (1) insulin-like growth factor 2 (Igf2), which is an autocrine factor involved in the proliferation of VSELs, and (2) RasGrf1, which is a GTP-exchange factor (GEF) crucial for signaling from activated insulin-like growth factor 1 receptor (IgfIR) and insulin receptor.^35^ In addition, hypermethylation of the DMRs on the maternal chromosome that encode Igf2R results in an additional negative effect on IIS in VSELs. As previously described, Igf2R serves as a decoy receptor that prevents Igf2 from binding to IgfIR.^36^ This epigenetic reprogramming of genomic imprinting negatively affects IIS signaling, maintains the quiescent state of murine VSELs (Figure 1), and, thus, protects VSELs from premature depletion from the tissues and prevents their involvement in tumor formation. Our most recent unpublished data on the methylation status of DMRs at the *Igf2-H19* locus in human VSELs suggest that a similar mechanism may also operate in human VSELs.

Next, based on the published studies reporting that IIS signaling negatively affects the lifespan of experimental animals,^37, 38^ we proposed a hypothesis that relates aging, longevity and IIS to the abundance and function of pluripotent VSELs that are present in adult murine tissues.^39^ Accordingly, we postulated that a prolonged IIS may negatively affect the pool of VSELs and, subsequently, TCSCs in various organs, thereby having an impact on tissue rejuvenation and lifespan.^33^ In support of this notion, we reported a significantly higher number of VSELs in BM of long-living murine strains of mice (for example, Laron and Ames dwarfs), whose longevity is explained by low levels of circulating IGF1 and, thus, a decrease in IIS.^40^ In contrast, the number of VSELs is reduced in mice with high levels of circulating Igf1 and enhanced IIS (for example, growth hormone-overexpressing transgenic mice) in comparison with normally aging littermates.^41^

We are currently investigating the influence of calorie restriction and the effects of the prolonged administration of drugs that modulate IIS and extend lifespan, such as metformin and rapamycin, on the pool of VSELs residing in BM and other tissues.

## Optimization of isolation strategies

VSELs are very rare (∼1 per 10^5^ monocular BM cells), and careful follow-up isolation protocols are required to purify these cells. Moreover, more specific markers are needed to isolate these cells. Nevertheless, at this point, the best positive surface markers that allow purification by multisorting analysis are the Sca1 antigen for murine VSELs and the CD133 antigen for human counterparts. However, these markers are also expressed by several other types of stem cells.

### Current strategies for isolating murine VSELs

The current strategy for purifying murine VSELs from BM, as well as other adult tissues, is based on sorting any nucleated cells that are slightly smaller than erythrocytes (4–5 μm) and express the Sca1^+^Lin^−^CD45^−^ phenotype. These cells also express SSEA1 antigen on their surface, and some VSELs also express receptors for FGF2 (FGF-2R), PDGF (PDGF-R), SDF1 (CXCR4) and KL (c-kit). We are aware that the lack of specific positive markers for VSELs leads to isolation by cell sorter of rare events that are enriched for VSELs but also contain some cell debris and even bare nuclei. Moreover, when isolating VSELs from murine BM, the most important factor is proper maintenance that consists of a cocktail of lineage antibodies and a sufficient titer against erythroid cells (Ter119) in order to avoid contamination of the sorted VSELs with Lin^−^CD45^−^ erythroblasts that may acquire low expression of Sca1 antigen by microvesicles shed from Sca1^+^ cells in BM. Thus, to avoid enrichment of the erythroblasts, it is also important to set up a proper gate that excludes small Sca1^dim^Lin^−^CD45^−^ cells, which could become contaminated by erythroblasts.

As mentioned above, some murine VSELs express FGF-2R, PDGF-R, CXCR4 and c-kit on their surface. We still do not know whether this expression of various receptors reflects the commitments of some VSELs to different lineages. In support of this possibility, we recently isolated three different types of mRNA from singly purified VSELs that differ in terms of the expression of pluripotency, germline-specific and various other types of genes.^42^

As murine BM is a relatively easy tissue for Fluorescence-activated cell sorting (FACS) analysis and cell sorting, most molecular data are obtained using BM-derived VSELs. However, as there are corresponding populations of cells in other murine solid organs,^43^ further work is needed to compare the molecular signatures of solid organ-purified and BM-isolated VSELs. Similar studies also have to be performed on human VSELs. Nevertheless, in a recent report, an independent group of investigators performed mRNA gene array analysis on human ovarian surface epithelium-derived VSELs and observed similar patterns of expression in genes regulating stem cell pluripotency and germ line specification,^44^ as we described in murine BM-isolated VSELs.^17^ Interestingly, these small human VSELs were efficiently sorted using FACS by employing antibodies against human SSEA4 antigen.

### Current strategies for isolating human VSELs

As described, VSELs from human UCB and mobilized peripheral blood were initially purified as small CD133^+^ CD45^−^ Lin^−^ cells by employing multiparameter sorting from an erythrocyte depleted by hypotonic lysis population of nucleated cells.^18^ Unfortunately, this isolation procedure is time consuming, and the sorting time required to process an entire unit of cord blood (∼50–100 ml), which is needed to isolate rare VSELs from mononucleated UCB cells, would be 3–4 days. Therefore, it has become clear that a faster and less expensive method for isolating these cells must be established. To develop a more efficient method for purifying VSELs from UCB, we proposed a three-step isolation strategy based on (1) removing erythrocytes by hypotonic lysis, (2) immunomagnetic separation of CD133^+^ cells, and (3) FACS-based isolation of CD133^+^Lin^−^CD45^−^ cells. Processing 100-ml UCB requires only 2–4 h using this procedure.^45, 46^ As with murine VSELs, we do not have a specific marker for VSELs, and our sorted cells may also contain other CD133^+^ cells. Furthermore, as with murine VSELs, it is very important to employ antihuman erythroid lineage antibodies (CD235a) to avoid contamination with small Lin^−^CD45^−^ erythroblasts.

As an alternative strategy, we also attempted exposing erythrocyte-depleted, immunomagnetic bead-selected CD133^+^ cells to Aldefluor, followed by staining with anti-CD133 antibodies conjugated with fluorochromes and two lineage-specific antibodies—one against pan-hematopoietic antigen CD45 (anti-CD45 MoAbs) and second one against erythroid marker glycophorin-A (anti-GlyA MoAbs).^47^ Aldefluor employed in this alternative sorting strategy is a substrate for aldehyde dehydrogenase (ALDH), a cytosolic enzyme that is highly expressed in less-differentiated hematopoietic cells. In the presence of ALDH, Aldefluor is modified to a fluorescent molecule that maybe used to mark ALDH-expressing cells. Using this novel strategy, we sort CD133^+^ cells enriched for VSELs and obtain from 100 ml of UCB, on average, ∼10^3^ CD133^+^CD45^−^GlyA^−^ALDH^low^ and 4 × 10^3^ CD133^+^CD45^−^GlyA^−^ALDH^high^ VSELs from 100 ml of UCB.^47^ These numbers demonstrate how rare these cells are in UCB. Moreover, when we compared both fractions of VSELs using RT-PCR, we found that CD133^+^CD45^−^GlyA^−^ALDH^low^ VSELs demonstrated a higher expression of the pluripotency marker Oct4 than the CD133^+^CD45^−^GlyA^−^ALDH^high^ fraction.^47^

However, we are aware that there is still room for improvement when it comes to sorting by employing, for example, metabolic fluorochromes to see whether VSELs are enriched among Rh123^dull^, Pyronin Y^low^ and Hoechst 33342^low^ cells. Moreover, we expect our proteomic data analysis of UCB-derived VSELs, which is in progress, will reveal the presence of new positive markers that could be employed for sorting these cells. However, as mentioned above, some groups have successfully employed antibodies against SSEA antigens to purify human^22^ and rat VSELs.^48^

Nevertheless, in order to further enrich human VSELs, we recently employed a novel strategy in which we deplete the lineage to thereby enrich CD133^+^ UCB-derived mononuclear cells, which are subsequently permeabilized and stained with anti-Oct4 antibodies. Using this strategy, we isolated a highly purified population of VSELs that was suitable for molecular studies at the DNA (imprinting analysis) and mRNA (gene expression analysis) levels (manuscript in preparation).

## The molecular signature of VSELs—the key to understand quiescence and pluripotency

As mentioned above, we performed most of our molecular studies on murine BM-purified VSELs at the DNA, mRNA and, to some extent, the protein levels.^31, 42, 49^ These DNA studies revealed that promoters for pluripotency markers, such as Oct4 and Nanog, have an open chromatin structure and are associated with transcription-promoting histones.^31, 49^ This finding strongly supports the authenticity of the expression levels of these pluripotency-regulating transcription factor genes in murine VSELs. Furthermore, as expected from the definition of PSC, we reported that VSELs possess so-called bivalent domains in developmentally crucial homeobox genes.^42^ As mentioned above, murine VSELs also possess a unique methylation pattern at the DMRs of some paternally imprinted genes (for example, erasure of imprinting at the *Igf2-H19* and *RasGrf1* loci and hypermethylation at the *Igf2R* and *KCNQ1* loci) that is responsible for the quiescent state of these cells (Figure 1) and the decrease in IIS. These changes in the expression levels of paternally imprinted genes were subsequently confirmed using mRNA analysis.^31^

Furthermore, our genome-wide analysis of murine BM-derived VSELs revealed that the attenuation of mitogenic growth factor signaling pathways also has a crucial role in quiescence and ageing.^50^ Specifically, VSELs downregulate the genes involved in the responses to UV radiation, mRNA processing and mitogenic growth factor signaling (for example, from Igf1 and TRKA receptors involved in the ERK and PI3K pathways). Using leading-edge subset analysis and real-time quantitative PCR assays, we observed that several genes, such as *Grb2, Sos1, Shc1, Map2k1, Akt3, Elk1, Rps6ka3, Gsk3β* and *Csnk2a1*, which are involved in mitogenic growth factor signaling pathways, are commonly downregulated in VSELs. In contrast, we observed that Oct4^+^ VSELs upregulate tissue-specific gene sets and a gene set that encodes the complement-coagulation cascade.

These results suggest that the epigenetic reprogramming of genomic imprinting maintains the quiescence of the Oct4^+^ epiblast/germ line-derived VSELs that are deposited in the adult body and protects them from premature ageing and uncontrolled proliferation.^51^ On the other hand, reversal of this mechanism will be crucial for employing VSELs as a population of PSCs for use in regenerative medicine. Currently, we are investigating how the downregulation of the expression of H19 enhances VSEL expansion, as recently demonstrated in parthenogenesis-derived PSCs.^52^

As briefly mentioned above, our mRNA expression studies revealed that VSELs express several epiblast and germline markers, which is the basis for the hypothesis that VSELs originate from early epiblast-derived migrating PGC-like cells. We envision that VSELs are deposited in adult tissues during development as a source of TCSCs and have a role in organ rejuvenation. In support of this notion, molecular analysis of murine BM-derived VSELs revealed that these cells express several genes that are characteristic of epiblast stem cells such as *Gbx2, Fgf5* and *Nodal* and germline specification of stem cells including *Blimp1, Prdm14, Fragilis, Stella, Nanos3* and *Dnd1*.^49^ Recently, we also found that both murine and human VSELs highly express Sall4, a marker of germline cells. We also demonstrated that murine and human VSELs (1) are diploid, (2) are viable, as shown by their ability to exclude viability dye (7-aminoactinomycin D), and (3) highly express telomerase.^17^

We are currently assessing the expression of various miRNA species in VSELs, as well as how to perform proteomic analysis on these rare cells. We hope the latter approach will allow us to identify new and unique markers for these cells that will allow optimal and rapid purification.

## Data supporting the presence of very small stem cells in adult murine and human tissues

As mentioned above, several primitive cells with the characteristics for pluripotency or multipotency were isolated from adult murine and human BM, UCB and adult solid organs, enzymatically processed using proteolytic enzymes, and expanded and cultured *in vitro*.^14, 53, 54, 55, 56^ From these cultures, in which the cells were allowed to grow and adhere to plastic or fibronectin, several populations of primitive cells were isolated, expanded and assigned different operational names such as MAPCs, MASCs, marrow-isolated adult multilineage-inducible cells, Muse cells or unrestricted somatic stem cells.^11, 12, 13, 14, 15, 16, 57^ Unfortunately, the phenotype of the stem cell that initiated these cultures has never been clearly described. Furthermore, very small cells, which are most likely human VSELs, can also be directly purified from neonatal UCB.^22, 58^ In addition to multiparameter sorting, other isolation strategies have been employed, and, for example, an interesting population of small cells that is able to differentiate into epithelial and HSCs was isolated from murine BM by elutriation, lineage depletion and the ability to home (H) to BM, has been described as ELH stem cells.^59, 60, 61^ Another group reported presence in adult mammalian tissues of small cells, which are able to differentiate into cells from all germ layers and have been isolated from adult mammalian tissues (known as ‘spore-like stem cells'). However, the purification strategy and expressed surface markers were not described in the original report.^62^ Nevertheless, the presence of ELH stem cells and ‘spore-like stem cells' residing in adult BM has somehow challenged the concept of HSC plasticity.

The most common features of VSELs are their very primitive morphology and relatively small size.^17, 18^ Recently, several reports were published that support the existence of small, primitive VSELs and VSEL-like cells in adult tissues (the most important are listed in Table 1). In addition to ELH cells and the spore-like stem cells mentioned above, VSELs or VSEL-like stem cells have been isolated by other independent groups. For example, murine BM-derived VSELs have been shown to give rise to type 2 pneumocytes, which produce lung surfactant protein after being transplanted into surfactant-deficient mice.^21^ Furthermore, small Oct4^+^SSEA1^+^Lin^−^CD45^−^ cells isolated from rat BM gave rise *in vivo* to cardiomyocytes and endothelial cells in an experimental model of rodent acute myocardial infarction.^48^ Moreover, cells from human-mobilized PB expressing the SSEA4^+^CD133^+^CXCR4^+^Lin^−^ and CD45^−^ phenotypes that were isolated using FACS formed human bones when embedded in gelatin sponges and implanted into immunodeficient mice; this bone-forming activity exceeded the activity of the other populations of BM-purified cells that were evaluated using the same assay. Based on this finding, it has been proposed that these PB-purified VSELs are at the top of the hierarchy for the mesenchymal and endothelial lineages.^23^ Cells similar to BM-derived VSELs have been reported to reside in the ovarian surface epithelium in postmenopausal ovaries^63^ and normal testes^64^ and differentiate into gametes.^44^ Furthermore, human PB-VSELs have been successfully purified by other researchers,^23, 65^ and very small Oct4^+^Sox2^+^ cells corresponding to UCB-derived VSELs described by us were purified from UCB by other investigators, who described them as a population of UCB-derived embryonic-like stem cells.^58^ Interestingly, these cells were able to differentiate *in vitro* into neural progenitor cells. Finally, a corresponding population of primitive Oct4^+^ stem cells that resembles VSELs—named omnicytes—was also described to be circulating in UCB and was capable of migrating into the maternal circulatory system.^66^

In addition to the cells listed in Table 1, in one of recent reports small cells with some of VSEL markers have also been identified in murine neonatal retina.^67^ In another study, small nonhematopoietic lineage-negative (Lin^−^) cells that have been isolated from adult BM by elutriation (Fraction 25 or Fr25) were involved in retinal regeneration following the induction of anterior ischemic optic neuropathy and optic nerve crush injury in a rodent model.^68^ Moreover, a similar population of small nonhematopoietic CD45^−^ stem cells harvested from BM via elutriation was recently shown to differentiate *in vivo* into functional insulin-producing cells in chemically induced diabetic mice.^69^ Finally, several features of VSELs are present in Muse cells that were recently isolated from murine and human BM populations.^16, 57^ As postulated by the authors, Muse cells may have a major role in populations of cells that preferentially become transformed and give rise to immortal induced PSCs when BM-derived stromal cells are induced to pluripotency by genetic manipulation. Thus, VSELs and VSEL-resembling cells are currently purified in several laboratories worldwide, and the coming years will bring more information regarding their biology and *in vitro* and *in vivo* differentiation potential.

## *In vitro* and *in vivo* criteria for stem cell pluripotentiality—comparison with VSELs

Several criteria have been proposed for classifying stem cell as PSCs (Table 2). These criteria were established by embryologists working on ESCs and induced PSCs. However, some of these stringent criteria of pluripotency listed in (Table 2), such as complementing blastocyst development and teratoma formation, are not always applicable for pluripotent epiblast stem cells (EpiSCs) as well as PGCs.^70, 71^

Our experimental data support that murine VSELs fulfill all the *in vitro* criteria listed in Table 2. In particular, as discussed in this review, murine VSELs not only possess the primitive morphology of early developmental cells (for example, high nuclear/cytoplasmic ratio, presence of euchromatin in the nucleus) but also express the marker characteristics for PSCs (for example, *Oct4, Nanog, Rex1*). Significantly, we recently confirmed the authentic expression of *Oct4* in murine VSELs by demonstrating that the Oct4 promoter have an open chromatin by direct analysis of methylation state (hypomethylation in DNA) and its high association with transcription-favorable histone modifications (for example, the acetylation of histone H3 and trimethylation of lysine 4 on histone 3) and relatively low association with the transcription-restricting ones (for example, dimethylation of lysine 9 on histone 3).^31^ We also studied the epigenetic state of another pluripotency-related transcription factor, Nanog. In comparison to Oct4, the promoter of Nanog has a higher level of methylation in VSELs (∼50%), and the quantitative chromatin immunoprecipitation experiments performed in parallel revealed that the H3Ac/H3K9me2 ratio favors transcription and maintains its active state.^72, 73, 74^ Based on these results, we conclude that murine VSELs truly express both *Oct4* and *Nanog*.

In addition, murine VSELs fulfill also other *in vitro* criteria of pluripotency (Table 2) such as presence of bivalent domains in promoters that encode developmentally important homeobox-containing transcription factors (*Sox21, Nkx2.2, Dlx1, Lbx1h, Hlxb9, Pax5* and *HoxA3*)^42^ and VSELs derived from female mice reactivate the X chromosome. Finally, we and other groups have successfully differentiated VSELs *in vitro* into cells from all three germ layers. Accordingly, recently published studies confirm that murine BM-derived VSELs can differentiate *in vivo* into HSCs,^47^ MSCs,^19^ endothelial cells,^48^ epithelial cells of the lung,^21^ oocytes,^44^ tumor stroma cells^29^ and cardiomyocytes.^20^

However, murine VSELs do not complete blastocyst development and do not develop teratomas, which are included in the *in vivo* criteria of PSCs (Table 2). This obvious discrepancy between the *in vitro* and *in vivo* PSC criteria for VSELs was recently explained by the effects of epigenetic changes in the expression of some paternally imprinted genes,^31^ as described above. Moreover, one has to take into consideration that, although all these *in vivo* criteria apply very well for ESCs and iPSc, they are not always applicable as mentioned above for EpiSCs and PGCs.

## Conclusions

New data from our and other independent laboratories have provided more convincing evidence for the existence and biological role of VSELs in murine adult tissues and their potential roles in (1) tissue organ rejuvenation, (2) regulation of lifespan, and (3) the regeneration/repair of damaged organs. We have to emphasize that, although the molecular nature of murine BM-derived VSELs has been extensively characterized, more research is needed to better characterize these small cells in humans. If we can confirm that a similar mechanism based on the epigenetic changes in somatic-imprinted genes operates in human VSELs, perhaps the controlled modulation of this imprinting state to produce proper *de novo* methylation of the regulatory regions in these genes on the maternal and paternal chromosomes could increase the regenerative power of these cells. This would allow for application of VSELs in clinical medicine. Finally, in order to avoid mistakes in isolation of VSELs we recommend for further reading our most recent paper^75^ in which we address in more details potential pitfalls in FACS sorting strategy. We also recommend an excellent recent review on VSELs published by Dr Krause group.^76^

## Figures and Tables

**Table 1:** Summary of the findings from a selection of reports on stem cells that are attributable to VSELs or closely related cells

*Cells originally named in the paper*	*Isolation procedure and properties described in the original paper*	*Reference*
ELH cells	Isolated by elutriation, lineage depletion and recovery after homing (H) to the BM. Differentiate to epithelial cells and hematopoietic cells.	^59, 60, 61^
Spore-like stem cells	Small, ∼5 μm in diameter, isolated from various murine tissues, resistant to freeze/thawing, express Oct4 and demonstrating broad differentiation. Isolation procedures not indicated.	^62^
Small nonhematopoietic Sca1^+^ Lin^−^ CD45^−^ cells	Isolated using FACS from murine BM, differentiate into type II pneumocytes, produce surfactant in the lung alveolar epithelium. Recently these cells were confirmed as VSELs.	^21^
Rat VSELs	Isolated using FACS from rat bone marrow as SSEA^+^ Lin^−^ CD45^−^ cells and endowed with cardiomyogenic and endothelial potential.	^48^
Human PB-derived VSELs	Isolated using FACS as SSEA4^+^ CD133^+^ CXCR4^+^ Lin^−^ and CD45^−^ cells, described as being at the top of the mesenchymal lineage hierarchy. Develop into human bones in immunodeficient mice.	^23, 65^
Ovarian VSELs	Small Oct4^+^ SSEA^+^ cells were isolated using FACS from ovarian surface epithelium (OSE) obtained from mice and humans (precursors of female gametes). Human OSE-derived VSELs were extensively characterized using mRNA expression array analysis.	^44, 63^
Testicular VSELs	Small Oct4^+^ SSEA^+^ cells identified in murine and human testes (precursors of male gametes).	^64^
Embryonic-like stem cells from UCB	Small CD45^−^/CD33^−^/CD7^−^/CD235a^−^ pluripotent stem cells (2–3 μm in diameter) coexpressing embryonic stem cell markers, including Oct4 and Sox2, able to differentiate into neuronal cells.	^58^
Omnicytes	Small Oct4^+^ stem cells identified in UCB, able to establish fetal–maternal chimerism.	^66^

Abbreviations: BM, bone marrow; FACS, 5 Fluorescence-activated cell sorting; UCB, umbilical cord blood; VSEL, very small embryonic-like stem cell.

**Table 2:** *In vitro* and *in vivo* criteria for pluripotent stem cells

	*VSELs*
*In vitro criteria for PSCs*	
Undifferentiated morphology, euchromatin and high nucleus/cytoplasm ratio	Yes
PSC markers (for example, Oct4, Nanog, SSEA), open chromatin at the Oct4 promoter, bivalent domains and reactivation of the X chromosome in female PSCs	Yes
Broad multilineage differentiation into cells from all three germ layers (meso-, ecto- and endoderm)	Yes
	
*In vivo criteria for PSCs*	
Complement blastocyst development	No
Teratoma formation after inoculation into immunodeficient mice	No

Abbreviations: PSC, pluripotent stem cell; VSEL, very small embryonic-like stem cell.
